# Impact of passive smoking on renal vascular morphology

**DOI:** 10.31744/einstein_journal/2022AO0011

**Published:** 2022-07-21

**Authors:** Carlos Alberto de Moraes, Bárbara Valtudes Nogueira Thal, Julia Veronese Bannwart, Regiane Aparecida Jacomini, Mercia Breda-Stella, Cesar Alexandre Fabrega Carvalho

**Affiliations:** 1 Faculdade de Medicina de Jundiaí Jundiaí SP Brazil Faculdade de Medicina de Jundiaí, Jundiaí, SP, Brazil.; 2 Faculdade de Santa Bárbara D’Oeste Jundiaí SP Brazil Faculdade de Santa Bárbara D’Oeste, Jundiaí, SP, Brazil.

**Keywords:** Tobacco smoke pollution, Endothelium, Tunica media, Adventitia, Collagen, Kidney cortex

## Abstract

**Objective:**

To determine whether passive smoking causes morphological and structural changes in the arcuate arteries of rats exposed for 7 to 28 days.

**Methods:**

Wistar rats aged eight weeks and weighing 260g on average were allocated to a Control or a Smoker Group. Groups were further divided into 4 groups containing 5 animals each. Morphological-functional analysis of the right kidneys was carried out after 7 and 28 days of exposure to the smoke of 40 cigarettes per day. Cigarettes were burned at set times using automated cigarette-burning equipment (“Smoking Machine” - SM-MC-01). At the end of each exposure period, the kidneys were dissected and submitted to histological processing for morphological and quantitative analysis.

**Results:**

Exposure to cigarette smoke for 7 days led to a decrease in inner vascular diameter. Decreased thickness of the vascular tunica media was observed after exposure for 28 days. Increased thickness of the tunica adventitia, increased total vascular wall thickness, increased total vascular diameter and qualitative increase in collagen deposition were observed. Vascular volume increased after 28 days of exposure.

**Conclusion:**

Passive smoking has a negative impact on renal vasculature.

## INTRODUCTION

Smoking is one of the most important risk factors for chronic non-communicable disease development. According to the National Cancer Institute (INCA - *Instituto Nacional do Câncer*), cigarette smoking accounts for 200 thousand deaths per year in Brazil and up to 5 million deaths worldwide.^(1,2)^ Epidemiological data also indicate that smoking is associated with the development of approximately 50 diseases and is directly related to several types of death from cancer. Smoking accounts for 85% of deaths from chronic lung disease, 30% of deaths from different types of cancer (lung, mouth, larynx, pharynx, esophagus, pancreas, kidney, bladder, cervix, stomach, and liver), 25% of deaths from coronary disease (angina and heart attack) and 25% of deaths from cerebrovascular diseases.^(3,4)^

Cigarettes contain thousands of chemical compounds (5,6000 according to recent estimates). Of these, approximately 158 have toxic properties and may cause environmental pollution when released into the atmosphere.^(5)^ According to Heiss et al.^(6)^ passive smokers inhale 10 to 100 times less smoke compared to active smokers. Still, severe cardiovascular damage may result in both cases. Nicotine is also associated with the onset and progression of many pathological conditions for some reasons, such as increased production of free radicals involved in oxidative stress, reduced nitric oxide synthesis and resultant impaired endothelial vasodilation, and stimulation of cell proliferation.^(7)^

In a study comparing family members of smokers (Control Group) and volunteers who were not exposed to cigarette smoke, Dülger et al.^(8)^demonstrated the kidneys are affected by active smoking, among other organs. However, recent studies reported exposure to cigarette smoke has significant toxic adverse effects on the kidneys and may induce significant morphological and functional changes, including reduced kidney size and cortical thickness, reduced glomerular diameter and density and decreased glomerular filtration rate.^(9)^

The kidneys are highly vascularized organs. Hence, smoking and circulating nicotine cause renal vascular changes. However, associations between passive smoking and renal morphological-functional changes induced by cigarette smoke exposure have not been fully elucidated. The investigation of such associations is the main goal of the current study.

## OBJECTIVE

To examine morphological and structural features of the renal microvasculature of rats exposed to passive smoking for 7 to 28 days.

## METHODS

### Animal care

Animals were handled and housed according to National and Institutional Guidelines for Animal Welfare established by the Brazilian College of Animal Experimentation (COBEA - *Colégio Brasileiro de Experimentação Animal*) and the National Council for Control of Animal Experimentation (CONCEA - *Conselho Nacional de Controle de Experimentação Animal*). Procedures involving animal use were analyzed and approved by the Animal Experimentation Ethics Committee of *Faculdade de Medicina de Jundiaí* (FMJ) (# 286/2015). Efforts were made to treat animals humanely by minimizing suffering and discomfort as well as the number of animals used.

### Study design

Experimental study with male rats (*Rattus norvegicus albinus*) aged 8 weeks and weighing 260g on average. Rats (n=20) were allocated to the Control Group (CG) or the Passive Smoker Group (SG). These were further divided into CG 7 days (n=5), SG 7 days (n=5), CG 28 days (n=5) and SG 28 days (n=5). Rats in both groups were kept in the FMJ vivarium from November 2015 to January 2016. Rats (two per box) were housed in separate rooms under the following conditions: room temperature of 22±2°C and light/dark cycle of 12 hours. Rats received filtered water (mL) and feed (g) *ad libitum* (Nuvilab^®^; energy, 339kcal/1418kJ 100.0%, carbohydrates 54g or 63.4%, protein 22g or 25.9%, lipids 4g or 10.6% - per 100g serving/percentage of total energy value). Feed and water intake were monitored throughout the experimental period.^(9)^

**Figure 1:**
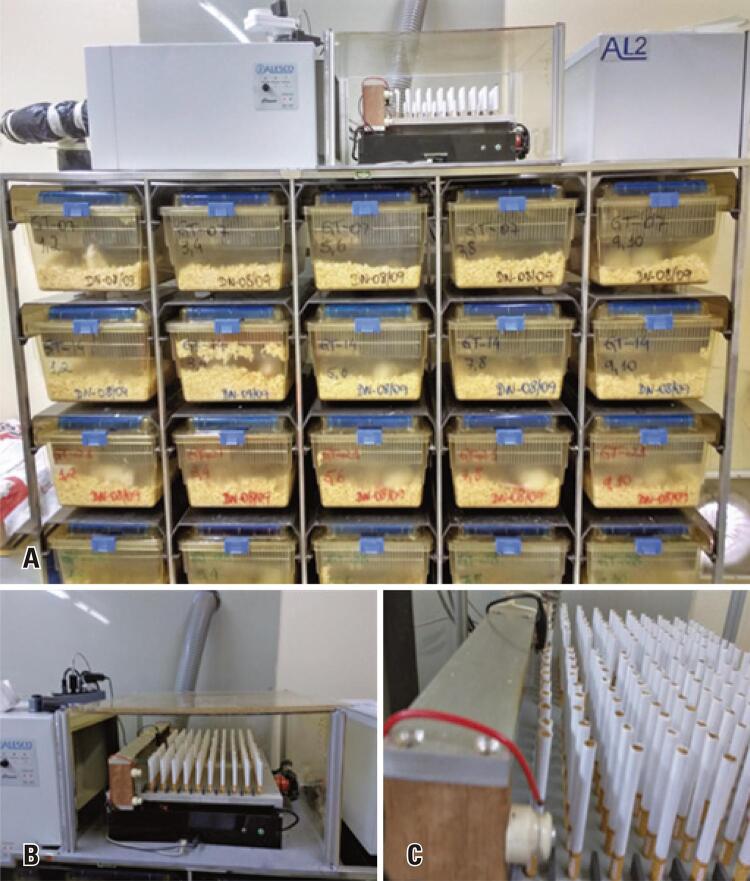
Air inlet of the ventilation system and smoking machine. (A) Panoramic view of the ventilation system (smoking room). Blower in the center of the smoking machine (top left) and exhaust fan (right). The back of the ventilation ducts and sealed boxes connected to the system (frontal plane) can also be seen; (B) Smoking machine containing 200 cigarettes and air inlet of the inflow system (left); (C) Detailed view of the smoking machine showing the resistance system for automatic cigarette burning

Source: Moraes CA, Breda-Stella M, Carvalho CA. Morphofunctional study on the effects of passive smoking in kidneys of rats. einstein (São Paulo). 2021;19:eAO6000.^(9)^

Adaptation of SG rats to cigarette smoke was achieved by gradual exposure to burning cigarettes. Rats were exposed in pairs to the smoke of up to 40 cigarettes per day (high yield; tar: 10mg; nicotine: 0.8mg; carbon monoxide: 10mg) for 7 to 28 days. Cigarettes were burned in an automated smoking machine (SM-MC-01), set to burn 10 cigarettes every 6 hours (4 cycles). Cigarette smoke was aspirated via the air inlet of the ventilation system (“smoking room”; Figure 1A and B) and homogeneously distributed in sealed SG rat boxes. Carbon dioxide was measured in CG and SG rat boxes and housing environment using a calibrated carbon dioxide meter (AZ Instruments, model AZ 77535, serial number 10109975) (certificate 00948/2016).^(9)^

### Kidney weight, size, and histology

On days 6 and 27 of the experimental period, rats were placed in a metabolic cage for 24-hour for urine collection for future analysis. Rats were then weighed, anesthetized and euthanized by prolonged anesthesia. The right kidney was harvested and weighed using an analytical scale (Mettler Toledo - Model AB204). Kidney length, width and height were measured using a caliper. The radius (r) was used to estimate kidney volume in cubic centimeters (cm^3^), according to the following ellipsoid volume formula: vol=4/3π (r1.r2.r3).^(9,10)^ For histological analysis, the right kidney was sectioned transversely between the upper and lower poles, embedded in paraffin and cut into approximately 4µm thick sections 80µm apart. Three histological slides from the kidneys of GC and GT rats were prepared and stained with hematoxylin-eosin (HE) and Picrosirius Red for morphometric analysis.

### Morphometry and stereology

Renal cortex measurements were obtained from HE-stained histological slides of the right kidneys using a calibrated (mm) eyepiece attached to a Motic optical microscope equipped with a 4X objective lens. Measurements were made at 5 different sites, as follows: upper and lower poles, at the level of the renal hilum and at midpole. Morphological and quantitative analysis of tissue structure was performed using Motic Images Plus 2.0 software for analysis and digitization of histological sections.

**Figure 2:**
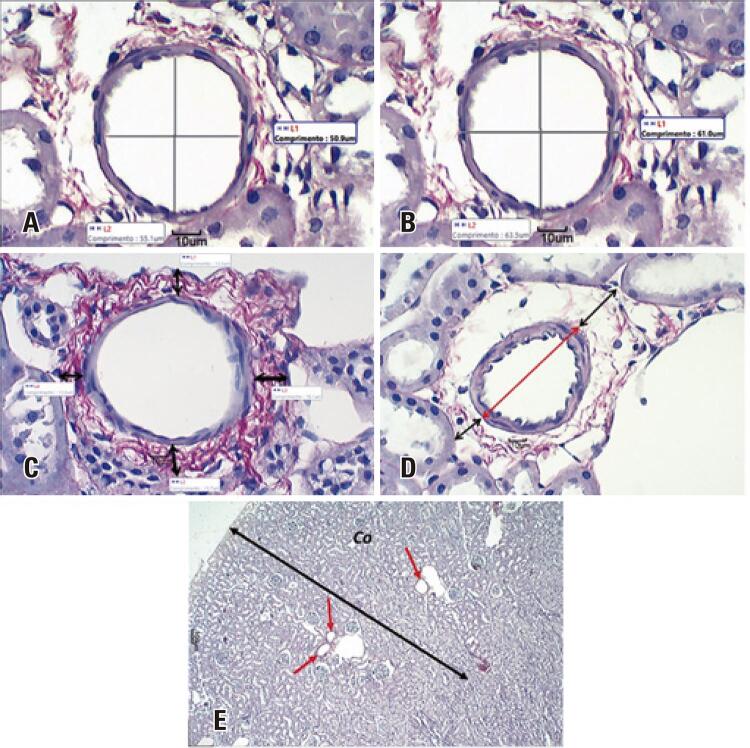
Photomicrograph measurements (40X magnification). (A) Inner vascular diameter (L1 and L2); (B) Outer vascular diameter (L1 and L2); (C) Arcuate artery tunica adventitia thickness (L1, L2, L3 and L4); (D) Total vascular diameter. The red line indicates the outer vascular diameter of the tunica media; black lines indicate the thickness of the tunica adventitia. Mean total vascular diameter is given by the sum of averaged measurements; (E) Arcuate artery volume density. Red arrows indicate arcuate arteries in the renal cortex (Co)

Cross-section images of the arcuate arteries of the right kidneys captured from histological slides stained with Picrosirius Red at 40X magnification were used for quantification of tunica media thickness. Lines were drawn between the ends of the arcuate arteries and values (µm) of outer and inner vascular diameter relative to the tunica media calculated by the software (Figures 2A and B). Tunica media thickness was calculated using the following formula: T.M.T = O.V.D - I.V.D (T.M.T = Tunica Media Thickness; O.V.D = Outer Vascular Diameter; I.V.D = Inner Vascular Diameter).

In the same vessels, lines were drawn at 4 cardinal points (east-west; north-south) to calculate the thickness (μm) of the tunica adventitia. Measurements were averaged and the mean value obtained (Figure 2C). Measurements were made under polarized light at 60X magnification. Collagen deposition was investigated. Mean outer tunica media diameter and mean tunica adventitia thickness values were added for total vascular diameter (μm) calculation (Figures 2D).

Arcuate artery volume density was estimated from histological slides by inspection and counting at 4X magnification (Figure 2E). Volume fractions occupied by arcuate arteries in the renal cortex were calculated using the following formula: Vv[vasc] (v/mm^2^) = nv/AC, where vascular volume (Vv[vasc]) is given in v/mm^2^, nv indicates the number of vessels in the cortex and AC corresponds to the cortical area in mm^2^. Kidney length radius (𝒓length) and cortical thickness values were used for cortical area (AC) calculation. The following mathematical formula designed to calculate the area of an ellipse was used: AC = 𝒓length X cortical thickness X π.

### Statistical analysis

Statistical analysis was performed for intergroup (CG *versus* SG) and intragroup comparisons. Variables were expressed as means and standard deviations. Data were submitted to analysis of variance (ANOVA). The assumption of homogeneity of variance was tested using the Turkey’s method for multiple comparisons. The level of significance was set at p≤0.05. Statistical analyses were performed using Biostat 5.3 software (*Instituto de Desenvolvimento Sustentável Mamirauá,* Belém, state of Pará, Brazil).

## RESULTS

### Morphometry and vascular volume density

Smoke group rats had significantly lower mean renal volume (cm^3^) and smaller cortical thickness (mm) at both time points studied (Table 1).

**Table 1:** Measurements of renal volume, cortical thickness, vascular diameter, vascular thickness and vascular volume density at 7 and 28 days

Variables	Groups	
	Control	Smokers
Kidney volume (cm^3^)		
7 days	1.15±0.08	0.84±0.11^†^
28 days	1.27±0.08	1.04±0.18^†^
Cortical thickness (mm)		
7 days	2.31±0.12	2.00±0.10^†^
28 days	2.36±0.13	2.10±0.15^†^
I.V.D (µm)		
7 days	62.8±3.1	58.8±4.3
28 days	62.3±7.7	59.0±4.7
O.V.D (µm)		
7 days	75.5±6.4	68.5±3.9^†^
28 days	76.4±8.3	70.9±6.1
T.M.T (µm)		
7 days	12.6±6.2	9.75±1.9*
28 days	14.2±2.0	11.9±3.0^†^
TAT (µm)		
7 days	15.5±2.2	27.9±4.7^†^
28 days	11.5±1.0	23.5±5.6^†^
TVT (µm)		
7 days	28.2±6.7	37.6±5.4*
28 days	25.6±2.2	35.4±8.4*
TVD (µm)		
7 days	106.7±7.6	124.3±12.0*
28 days	99.4±9.5	117.9±14.8*
Vv[vasc] (v/mm^2^)		
7 days	0.43±0.02	0.40±0.05
28 days	0.30±0.02	0.44±0.07*

* differ by 5% in significance (p≤0.05); ^†^ differ by 1% in significance (p≤0.01). Results expressed as mean ± standard deviation derived from inter and intragroup analyses.

I.V.D = Inner Vascular Diameter; O.V.D = Outer Vascular Diameter; T.M.T = Tunica Media Thickness; TAT: tunica adventitia thickness; TVT: total vascular thickness; TVD: total vascular diameter; Vv[vasc]: vascular volume density.

**Figure 3:**
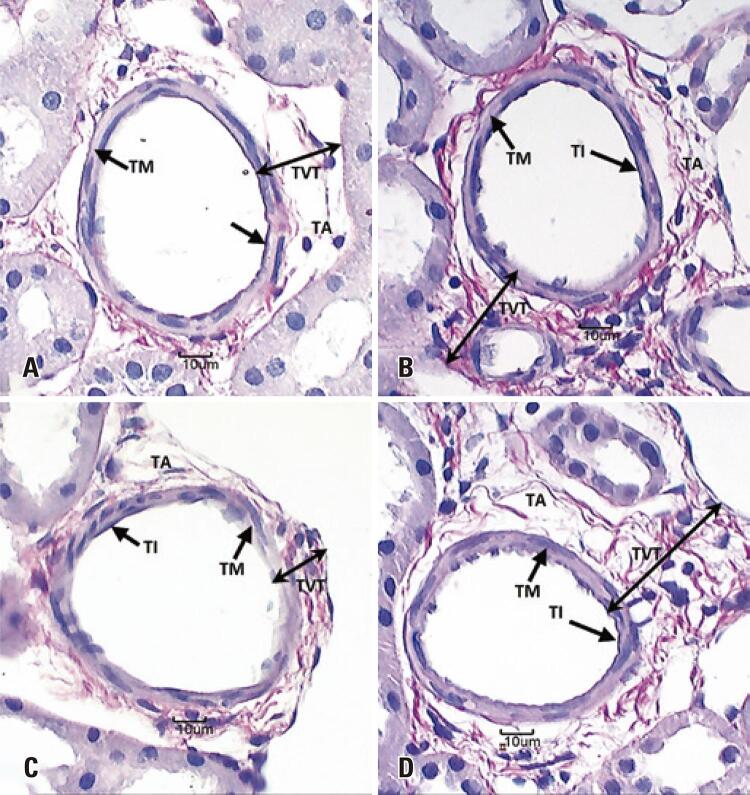
Photomicrographs showing the arcuate arteries of the right kidney. (A and C) Control Group. (B and D) Smoker Group. (A and B) 7-day period. (C and D) 28-day period

Mean inner vascular diameter (µm) remained unchanged over the course of the experimental period. However, mean outer vascular diameter (µm) was significantly smaller in rats exposed to cigarette smoke for 7 days) (Table 1 and Figure 3).

Note the tunica intima (TI), tunica media (TM) and tunica adventitia (TA). Note increased total vascular thickness (TVT) in rats in the Smoker Group. Picrosirius red staining, 40X magnification.

**Figure 4:**
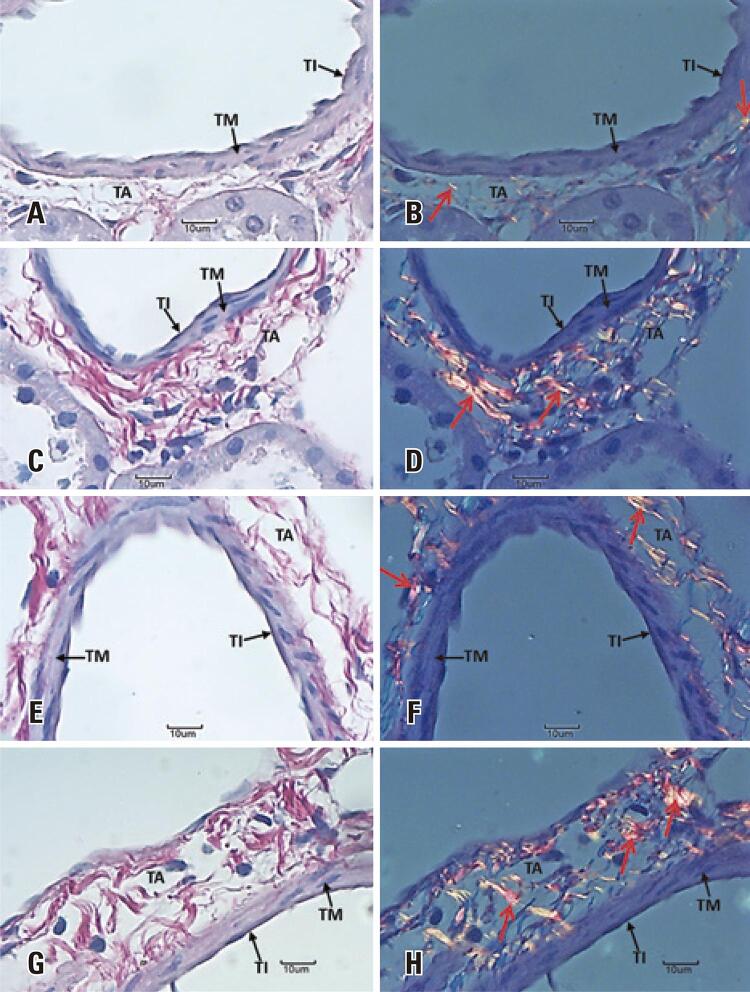
Photomicrographs showing the arcuate arteries of the right kidney. (A, B, E and F) Control Group. (C, D, G and H) Smoker Group. (A, B, C and D) 7-day period. (E, F, G and H) 28-day period

Mean tunica media thickness (µm) was significantly smaller in SG rats. Mean tunica adventitia thickness (µm), mean total vascular thickness and mean total vascular diameter (µm) were larger in these rats throughout the experimental period (Table 1 and Figure 3). Likewise, qualitative analysis of collagen deposition revealed increased deposition in the tunica adventitia at both time points studied (Figure 4). Mean vascular volume density (Vv[vasc] - (v/mm^2^)) was significantly higher in rats in rats exposed to cigarette smoke for 28 days (Table 1 and Figure 3).

Note the tunica intima (TI), tunica media (TM) and tunica adventitia (TA). Note increased collagen deposition in the TA (red arrow) of ratsin the Smoker Group. Picrosirius red staining, 40X magnification.

## DISCUSSION

Products from cigarette burning released into the environment are associated with a greater risk of morbidity and mortality.^(11)^Hence, smoking affects the health of both active and passive smokers. Exposure to cigarette smoke (equivalent to 1% of 20 actively smoked cigarettes per day) is thought to impair endothelial vasodilator function, leading to atherosclerotic disease.^(12)^

The most common vascular changes detected in smokers are fibroelastic tissue proliferation and thickening of the tunica intima in arterioles of organs that do not come into direct contact with cigarette smoke, such as the kidneys.^(13,14)^It has been well established in the literature that passive smoking may cause endothelial dysfunction.^(8)^ Culhaci et al.^(14)^ detected morphological changes in the kidneys of rats exposed to cigarette smoke and concluded that harmful effects on vessel walls are exposure time- and age-dependent. They also concluded that the kidneys of subjects with previous kidney injury are more prone to cigarette smoke-related injury due to pre-existing glomerular inflammation.

In this study, vascular wall thickness and total vascular diameter were significantly larger in SG rats throughout the experimental period. Similar findings were reported by Ferrer et al.^(15)^ In that experimental study,^(15)^ thickening of pulmonary artery walls in animals exposed to cigarette smoke was attributed to smooth muscle cell proliferation and elastic fiber deposition, with resultant increase in the total vascular thickness. Likewise, in a study published by Oberai et al.^(16)^ increased collagen deposition and smooth muscle cell proliferation were thought to account for the significant thickening of the myocardial and renal arteriolar walls observed in smokers.

However, the increase in total vascular diameter observed in SG rats in this study was primarily due to significantly increased thickness and collagen deposition in the tunica adventitia. This finding may have reflected a reactive cleavage mechanism of the adventitia, in which fibroblasts stimulated by pro-inflammatory agents synthesize new collagen to repair the structure of this tunica.^(17)^ In the literature, smoking is defined as an aggressive agent that promotes tissue injury and recovery via deposition of type III collagen.^(18)^ These factors have also been highlighted in studies with smokers carried out by Orth et al.^(19)^ Aside from proliferation of smooth muscle cells in renal arteries, increased collagen deposition in the tunica adventitia was reported in that study.^(19)^

According to Carty et al.^(20)^ in addition to endothelial smooth muscle cell proliferation, nicotine also enhances the expression of several extracellular matrix metalloproteinases involved in cell migration and remodeling of endothelial layers, such as collagenase-1, stromelysin-1 and gelatinase. The increased thickness of the tunica adventitia may reflect greater platelet adhesion to the endothelium in response to changes in the mechanism of platelet aggregation in animals exposed to cigarette smoke. Pittilo et al.^(21)^ suggested the expression of platelet-derived growth factor in vessel walls may induce connective tissue proliferation, leading to thickening of the tunica adventitia.

Increased vascular wall thickness due to smoking-related thickening of the tunica intima and tunica media has been well established in the literature. In their studies, Wright et al.^(22)^ observed that smoking may cause remodeling of the tunica intima and tunica media of vessels in response to inflammation induced by vasoactive substances and proteolytic enzymes. However, no significant increase in the tunica media or the tunica intima was observed in the SG rats in this study, probably due to short exposure time.

Vascular density increased significantly in rats exposed to cigarette smoke for 28 days. Literature data show that smokers tend to have higher vascular density due to endothelial lesions caused by exposure to cigarette smoke. Findings of clinical and experimental studies support the theory that angiogenesis occurs in the adult kidney and plays an important role in tissue regeneration in the presence of kidney injury.^(23,24)^ In *in vitro* studies, Lee et al.^(24)^ demonstrated that tissue and plasma concentrations of nicotine similar to those measured in light to moderate smokers induce pathological angiogenesis. Nicotine binds to endothelial acetylcholine receptors, promoting endothelial cell migration and proliferation and increasing nitric oxide production, with similar effects to other angiogenic growth factors. These findings suggest the participation of an angiogenic component in the pathophysiology of major smoking-related diseases, such as carcinoma and atherosclerosis. According to authors of that study,^(24)^ vascular changes and endothelial dysfunction induced by circulating nicotine and cotinine impair the self-regulatory capacity of the kidney, increasing the vulnerability of this organ to ischemic injuries.^(25)^ Hence, changes in vascular diameter, thickness and density observed in this study are in keeping with the data found in the literature.

## CONCLUSION

The findings of this study revealed that the exposure and passive inhalation of cigarette smoke by the animals allowed morphological and structural changes in the diameter, thickness and vascular density, as well as in the collagen deposition in the tunica adventitia and corroborate that exposure to passive smoking it can compromise renal function, with effects similar to those described in the literature in relation to active smoking.
